# List of Reviewers 2024 – 2025

**DOI:** 10.1017/S1368980026102407

**Published:** 2026-04-13

The Editorial Board of *Public Health Nutrition* would like to thank the following for their contribution as peer reviewers in 2024 and 2025:

Mirna Al Masri

Samuele Laudani

Anne Løvhaug

Noushin Mohammadifard

Cagla Ayer

Inger Aakre

Mahdieh Abbasalizad Farhangi

Safura Abdool Karim

Sofonyas Abebaw

Frances Aboud

Aylin Acikgoz Pinar

Rachel Acton

Habila Adamou

Kehinde Adeosun

Amaha Adhanu

Theodosia Adom

Aditika Agarwal

George Agogo

Luz Adriana Aguirre

Carlos Aguirre-Nunez

Marjan Ajami

Christelle Akl

Huda Al Hourani

Jaclyn Albin

Dania Aleman

Veruska Alexandre-Weiss

Omer Alkan

Dalal Alkazemi

Louise Allen

Stephen Allen

Luciene Fátima Almeida

Arlene Alpha

Mohammed Alsherbiny

Adriana Alvarado

Carlos Alves Junior

Ramya Ambikapathi

Bony Amin

Mary Amoako

Kim Anastasiou

Craig Anderson

Tatiana Andreyeva

Angeliki Angelidi

Donato Angelino

Cerra Antonacci

Valentina Antoniotti

Smaragdi Antonopoulou

Jessica Appleton

Katherine Appleton

Arman Arab

Sabrina Arasimowicz

Carolina Araya

Gastón Ares

Erik Kristoffer Arnesen

Joanne Arsenault

Sedat Arslan

Richmond Aryeetey

Yuka Asada

Dilek Aslan

Chloe Astbury

Mona AuYoung

Rasmi Avula

Lidia Bezerra Barbosa

Ka Brown

Lena Bababeer

Esther Babirekere-Iriso

Laxman Bablani

Tulay Bagci Bosi

Danielle Baird

Rosalie Bakker

Daniel Bandoni

Hasan Al Banna

Emmanuel Barankanira

Thomas Baranowski

Mayra Barata

Josep Xavier Barber Valles

Liza Barbour

Lorenzo Barnaba

Susan Barr

Eden Barrett

Maria Luisa Barros

Paloma Barros

Samantha Batchelor

Marieke Battjes-Fries

Micheline Beaudry

Andrea Begley

Bethany Bell

Philippe Belmont

Katerina Belogianni

Annemarie Bennett

Gabrielle Bennett

Rebecca Bennett

Alejandra Benítez-Arciniega

Aravinda Berggreen-Clausen

Resom Berhe

Carissa van den Berk-Clark

James Berkley

Jennifer Bernal Rivas

Meredith Bessey

Alejandra Betancourt-Núñez

Joline W. Beulens

Ilana Nogueira Bezerra

Dhiman Bhadra

Zulfiqar Bhutta

Chongjun Bi

Sander Biesbroek

Galya Bigman

Amelia Bird

Catherine Birken

Jennifer Black

Maureen M. Black

Rachel Bleiweiss-Sande

Jesús Blesa Jarque

Lauren Blum

Lisa Bodnar

Alexandra M. Bodnaruc

Eduarda Bogea

Leonie Bogl

Elias Bojago

Kristy Bolton

Daniela Bonofiglio

Leon Booth

Sue Booth

Camila Borges

Rafael Borneo

Christelle Bou-Mitri

Vasiliki Bountziouka

Abdellatif Bour

Emma Boyland

Çiğdem Bozkır

Patrick Brady

Cátia Braga-Pontes

Joana Brandao

Anne Lise Brantsæter

Didier Brassard

Tanya Braune

Barbara Brayner

Gemma Bridge

Julie K. Brimblecombe

Flávia dos Santos Brito

Christine Brombach

Diana Brostow

Louise Brough

Rachel Brown

Jennifer Browne

Janine Bruce

Maria Bryant

E. Jean Buckler

Nassib Bueno

Jose Luis Vazquez Burguete

Sarah Burkhart

Jennifer Burney

Charles Bitamazire Businge

Anne Byrne

Katarina Bälter

Funda Pinar Cakiroğlu

Thaís Cristina Marquezine Caldeira

Eric Calloway

Daniela Canella

Caroline Capitani

Marco Capocasa

Felipe Cardoso

Rachel Carey

Julia Carins

Monica Carlsen

Jessica Cartwright

Caitlin Caspi

Barabara Castillo

Caroline Tianeze de Castro

Bess Caswell

Esra Cataltepe

Laura Caulfield

Gustavo Cediel

Florencia Cessani

Raja Chakraborty

Rishika Chakraborty

Catherine Chan

Cindy Mei Jun Chan

Dian Chandra

M. Chaparro

Alice Chaplin

Emma Chappell

Karen Charlton

Preeti Chaudhary

Gabriela Chaves

Irazu Yanaina Chavez-Ugalde

Leila Cheikh Ismail

Bogale Chekole

Lanlan Chen

Wei Chen

Gwen Chodur

Nuzhat Choudhury

Mohammad Rocky Chowdhury

Aaron K. Christian

Ock Chun

Alexandra Chung

Christopher Cifelli

Joanne Clarke

Rafael Claro

Christine Cleghorn

Julianne Clina

Juliana Cohen

Elif Coker

Zoé Colombet

Uriyoán Colón-Ramos

Shannon Conrey

Alejandra Contreras Manzano

Rana Conway

Kirsten Coppell

Laura Cornelsen

Teresa Correa

Elizabeth Corrêa

Cintia Costa

Diogo Costa

Joel C Craddock

Gundersen Craig

Catherine Crespi

Simone Crouch

Jennifer Crowley

Katherine Cullerton

Diana Cunha

Kenda Cunningham

Laura D’Addezio

Karen D’Alonzo

Aline D’Angelo Campos

Heather D’Angelo

Ninoshka D’souza

Emre Duman

J. P. Dadhich

Fidelia Dake

Elnaz Daneshzad

Caitlin Daniel

N. Darmon

Fatemeh Darsareh

Isabel David

Marc De Buyzere

Marta De La Flor Alemany

Augusto Cesar De Moraes

Michelle DeBiasse

Yunyang Deng

Emily Denniss

Alexandra Descarpentrie

Kathryn Dewey

Preeti Dhuria

Mostafa Dianatinasab

Mariane Dias

Devilal Dimple

Sanne Djojosoeparto

Natalie Doan

Leonice Doimo

David Doledec

Grant Donnelly

Marie-Claude Dop

Samocha-Bonet Dorit

Pauline Douglas

Azam Doustmohamadian

Catherine Draper

Nine Droog

Valerie Duffy

Theogene Dusingizimana

Pratibha Dwarkanath

Duah Dwomoh

Johanna Dwyer

Louise Dye

James Dyer

Portia Dzivenu

Kübra Damla Ekenci

Shona Eagles

Creswell Eastman

Regina Eddie

Caroline Edmonds

Nilson Eduardo

Caitlyn Edwards

Tobias Effertz

Gudina Egata

Audrey Elford

Charlene Elliott

Shandell L. Elmer

Sarah Elshahat

Jennifer Emond

Carla Enes

Katherine Engel

Francisco Entrena Duran

Maijaliisa Erkkola

Nesli Ersoy

Nilay Etiler

Patricia Eustachio Colombo

Oluchi Ezekannagha

Pam Factor-Litvak

Olusegun Fadare

Shiva Faghih

Shah Mohammad Fahim

Umi Fahmida

Alexandre Faisal

Jennifer Falbe

Rosalind Fallaize

Ai-ping Fang

Albert Farre

Penny Farrell

Dominic Farsi

Mathias Fasshauer

Linda Ng Fat

Tera Fazzino

Judhiastuty Februhartanty

Rebecca Feicht

Fentaw Wassie Feleke

Ana Paula Fernandes Gomes

Ana Fernandes

Lara Fernandes

Sylvia Fernandez-Rao

Mackenzie Ferrante

Alberto Ferrarese

Marika Ferrari

Aline Ferreira

Fabrícia Ferreira

Nathália Ferreira

Barbara Fielding

Barbara Fiese

Roger Figueroa

Amy Finlay

Jordie Fischer

Valerie Flax

Catharine A. K. Fleming

Sondos Flieh

Pilar Flores

Maryah Fram

Sarah Francis

Kylie Fraser

Darcy Freedman

Laurence Freedman

Riitta Freese

Sophie Freije

Jan Friesinger

Edward Frongillo

Sara Frödén

Lydia Furman

Kamila Gabe

Cristina Gago

Elise Gahan

Jamie Gahche

Pablo Gaitán-Rossi

Robert Gajda

Ana Maria Gal

Danielle Gallegos

Francesca Galli

Mohd Ashraf Ganie

Kim Gans

Giselle Garcia

Marta García Sierra

Emma Garnett

Elisabeth Garratt

Tatjana Gazibara

Seifu Gebreyesus

Fuchs George

Alia Ghoneum

James Gibb

Ignacio Manuel Giménez-Alba

Karen Glanz

Deborah Gleeson

Yvonne Goh

Zeynep Goktas

Mahdieh Golzarand

Alicia Gomes

Clara Gomez-Donoso

Enrique Gomez-Pomar

Maria do Rosário Gondim

Ana Laura Gonzalez

Ines Gonzalez-Casanova

Sarah Gonzalez-Nahm

Wendy González

Cristina González-Díaz

Barry Goodwin

Wendi Gosliner

Megan L. Gow

Pamela Graham

Fernanda Granado

William Grant

Amanda Grech

Christian Gregory

Jessica Grieger

Anne Griffin

Quinn Grundy

Pauline Gulliver

Carolyn Gunther

Xu Guang Guo

Aakriti Gupta

Adyya Gupta

Klara Gurzo

Befikadu Gutema

Joanne Guthrie

Adane Habtie

Amir Hadi

Luc L. Hagenaars

Erin R. Hager

Fahimeh Haghighatdoost

Jess Haines

Marissa Hall

Lotte Hallez

Megan L. Hammersley

Rhona Hanning

Carolina Hargous

Lisa Harnack

Hasnah Haron

Kaitlyn Harper

Rachel Harris

Katherine Harris-Lagoudakis

Anna Harton

Kenneth Harttgen

Rebecca Hasdell

Nancy Jean Haselow

Mona Hashim

Saima Hasnin

Gonzalo Hatch-Kuri

Daniel Hatfield

Nguyen Thi Thu Hau

Hanna Hauptmann

Benjamin Hawkins

Nicola Hawley

Emma Haycraft

Frank Hayford

Ashleigh Haynes

Gabby Headrick

Gabby Headrick

Eva Heeremans

Samantha Heerschop

Archana Hegde

Azita Hekmatdoost

Christine Helle

Ursula Hemetek

Piril Hepsomali

Allison Hepworth

Ivonne Hernandez

Kirsten Herrick

Manjula Hettiarachchi

Bettina Hieronimus

Erin Hill

Jillian Hill

Frances Hillier-Brown

Anne Himberg-Sundet

David Himmelgreen

Patrícia Hinnig

Hilal Hizli Guldemir

Carmen Ho

Erin Hobin

Karen Hock

Jody Hoenink

Lisa Hofmann

Emily Hohman

Clare Holley

Kelseanna Hollis-Hansen

Graham Horgan

Omer Horovitz

Ana Horta

Susan Horton

M. Mahbub Hossain

Somayeh Hosseinpour-Niazi

Bailey Houghtaling

Amber Hromi-Fiedler

Kuen-Chang Hsieh

Sophia Hua

Qiushi Huang

Jorg Huber

Oliver Huse

Misba Hussain

Joy M. Hutchinson

Nahla Hwalla

Alessandro Iellamo

Iris Iglesia

Teresa Iglesias

Ozlem Ipek

Robin Ireland

Xavier Irz

Sejla Isanovic

Sebastian Isbanner

Kazuko Ishikawa-Takata

Theocharis Ispoglou

Rita Itani

Suvi Itkonen

Jana Jabbour

Inarie Jacobs

Laurie Jacobs

Harriet Jager-Wittenaar

Shirley James

Kathryn Janda-Thomte

Megan Jarman

Renuka Jayatissa

Agnes Jensen

Julie Jesson

Mahsa Jessri

Angeline Jeyakumar

Jie Jia

Si Si Jia

Qianxia Jiang

Jane Jih

Stephanie Jilcott Pitts

Maud Joachim-Célestin

Abigail Johnson

Alexandra Johnstone

Manuela Jomori

Catrin Jones

Maria Eduarda José

Petur Juliusson

Ju Young Jung

Leyla Karaoglu

Krishna Rao

Niina Kaartinen

A. F. M. Iqbal Kabir

Gilberto Kac

Henry S. Kahn

Emiliano Kakisu

Naomi Kakoschke

M. Kamruzzaman

Noora Kanerva

Ridhima Kapoor

Mickael Karlsson

Nancy Karreman

Caroline Karugu

Tilakavati Karupaiah

Christina Kasprzak

Thomas Kastner

Katsuhiko Kato

Mio Kato

Kendra Kattelmann

Michelle W. Katzow

Simrat Kaur

Justine Kavle

Enola Kay

Matthew Kearney

Jemma Keat

Aweke Kebede

Chee Cheong Kee

Matthew Keeble

Natalie Keirns

Matthew Kelly

Niamh Kelly

Joya Kemper

Aileen Kennedy

Lynne Kennedy

Katherine Kent

Marko Kerac

Jean Kerver

Viktorija Kesaite

Elina Kettunen

Seema Khadka

Mohammad Khalili

Hafiz Khan

M. Khan

Naiman Khan

Manorama Khare

Mairead Kiely

Valerie Kilders

Jung Eun Kim

Kirang Kim

Joel Kimmon

Sue Kleve

Linda Knol

Melissa Knox

Bennur Koca

Stephen Kodish

Nelene Koen

Dasa Kokole

Eda Koksal

Jane Kolodinsky

Yoko Komada

Joel Komakech

Hamed Kord Varkaneh

Małgorzata Kowal

Michael Krawinkel

Elizabeth Kristjansson

Jaclyn Kropp

Akihiro Kuma

Neha Kumar

Satyajit Kundu

Sarka Kunzova

Tony Kuo

Edye Kuyper

Laura König

Süleyman Köse

Elisa Maria Lacerda

Carl Lachat

Anthony Lacina

Chih-Cheng Lai

Amar Laila

Hannah Lambie-Mumford

Ginny Lane

Silvia Lapertosa

Matthew Lapierre

Nicole Larson

Fiona Lavelle

Carl Lavie

Thomas Lawler

Mark Lawrence

Deana Leahy

Tashara Leak

Gwenyth Lee

Jennifer Lee

Jung Eun Lee

Megan Lee

Rebekka Lee

Seung-Yeon Lee

Vivian Y. Lee

Yen-Han Lee

Rebecca Leech

Mira Lehberger

Anna Leibinger

Maria Leite

Herman Lelieveldt

Marcell Leonario

Lucia Leone

Jennifer Lerman

Jef Leroy

Lily Arsanti Lestari

Gloria Leung

Douglas E. Levy

Emma Lewis

Meron Lewis

Bai Li

Zhen Li

Rafaela Liberali

Nanna Lien

Juliana Lignani

Kuang Hock Lim

Chung-Ying Lin

Wei-Ting Lin

Xiao Lin

Amanda Linares

Rebecca Lindberg

Anna Lipert

Natalie Lister

Bochu Liu

Kai Liu

Ruru Liu

Shaojie Liu

Zhao-Min Liu

Zhiwei Liu

Margarida Liz Martins

Lindsey Locks

Stacy Loeb

Barbara Lohse

John Long

Rachel Loopstra

Elisama Lopes

Gabriela Wünsch Lopes

Tais Lopes

Esther Lopez-Garcia

Penelope Love

Caitlin Lowery

Marcos Anderson Lucas da Silva

Ruth Lucas

Angie Luna Pinzon

Thomas Lund

Leslie Lytle

Nancy López-Olmedo

Anne Lene Løvhaug

Brian Maugo

Özge Mengi Celik

Priscila Pereira Machado

Sally Mackay

Ahmed Madar

Damian Maganja

Catherine Mah

Mohammad Imteaz Mahmud

Taciana Maia de Sousa

Matthieu Maillot

Madhura Maiya

Hazreen Majid

Maishahataba Solomon Makwela

Maryam Maleki

Akbar Malekshah

Anita Malhotra

Shelly Malik

Mashudu Manafe

James Manley

Elpis Mantadakis

Dirce Marchioni

Oonagh Markey

Grace Marquis

Bernadette Marriott

Alison Marshall

Sarah Martinelli

Jose Martines

Angela Martinez

Homero Martinez

Sonia Martinez

Suzanna Martinez

Ana Paula Martins

Maria Rosario Martins

Lucile Marty

Juan de Toro Martín

Melvin Barrientos Marzan

Rainier Masa

Nester Mashingaidze

Juliana Matos

Lama Mattar

Nathalia Matties Maas

Tara Maudrie

Giuseppe Maulucci

Mohsen Mazidi

Mary McCann

Julia McCartan

Elaine McCarthy

Amanda McClain

Jo McCormack

Lacey McCormack

Megan McCrory

Lynn McIntyre

Fiona McKay

John McKenzie

Rachael McLean

Jennifer McMullen

Fernanda Mediano Stoltze

Sarah Meiklejohn

Juliana Mello

Yarisbel Melo Herrera

Alida Melse-Boonstra

Hannah Melville

Cansu Memiç Inan

Larissa Mendes

Raquel Mendonça

Reci Meseri

Biljana Meshkovska

Melissa Mialon

Georgia Middleton

Jochen Mierau

Kozeta Miliku

Amy Millen

Daniel Miller

Margaret Miller

Leia Minaker

Jonatan Miranda Gómez

Parvin Mirmiran

Lourence Misedah-Robinson

Diane Mitchell

Yoshihiro Miyake

Siluleko Mkhize

Shooka Mohammadi

Fatemeh Mohammadi-Nasrabadi

Masoud Mohammadnezhad

Zalilah Mohd Shariff

Panchali Moitra

Yoan Molinero-Gerbeau

Chika Momoki

Dorothy Monica

Pablo Monsivais

Luana Monteiro

Makama Monyeki

Amy Moore

Seyedeh Parisa Moosavian

Guennouni Morad

Sajjad Moradi

Alcides Morais Junior

Gladys Morales

Nancy Moran

Judy More

Kevin Morgan

Belinda Morley

Taryn Morrissey

Rebecca Mozaffarian

Sylvie Mrug

Magdalena Muc

Megan Mueller

Olivier Mukuku

Joshua Muliira

Angela Mulligan

Efren Murillo-Zamora

Marie Murphy

Sandra Murray

Aviva Musicus

Mira Mutiyani

Júlia Muñoz-Martínez

Ágota Mészáros

Beatriz Nunes

Lydiane Nabec

Andy Naja-Riese

Martha Nakakande

Mieko Nakamura

Mahdi Nalini

Janandani Nanayakkara

Nihal Natour

Shaan Naughton

Santiago Navas-Carretero

Hala Nawaiseh

Jabulani Ncayiyana

Denise Ndlovu

Leendert Nederveen

Sanja Nel

Michelle Nelson

Nicklas Neuman

Felipe Neves

Henry Ng’ethe

Eric Ng

Ha Nguyen Thu

Bong Nguyen

Mary Nicolaou

Claudia Nieto

Eduardo Nilson

Yanhua Ning

Morimoto Nobuhisa

Essra Noorwali

Nayely Noriega

Jennifer Norman

Amy Nunn

Danielle Nunnery

Georgina Núñez Rocha

Karen O’Callaghan

Kirstie O’Neill

Aaron J. O’Sullivan

Akira Obana

Feyisayo Odunitan-Wayas

Yukiko Okami

Elochukwu Okanmelu

Joshua Okyere

Beatrice Olack

Carol Oladele

Deborah Olarte

Florencia Olivares

Elizabeth Onyango

Yasmin Beng Houi Ooi

Laura Oostenbach

India Ornelas

Colin J. Orr

Jemma Orr

Dania Orta-Aleman

Ana G. Ortega-Avila

Hibbah Osei-Kwasi

Alireza Osteadrahimi

Richard Otwey

Ousmane Ouedraogo

Cecilia Isabel Oviedo-Solís

Elif Esra Ozturk

Sinéad O’Mahony

Mohammad Reza Pakravan-Charvadeh

Miranda Pallan

Philip Palmedo

Wen-Harn Pan

Morgan Pankhurst

Anushriya Pant

Angeliki Papadaki

Zacharias Papadakis

Ionas Papassotiriou

Marie-Claude Paquette

Sherly Parackal

Bunga Paramashanti

Hyejoon Park

Kyong Park

Jennie Parnham

Stephanie Partridge

Dori Patay

Divya Patel

Emma Patterson

Julie Patterson

Mari Mohn Paulsen

Denise Payan

Jo Pearce

Alexandra Pepetone

Federica Perazza

Giselle Pereira Pignotti

Alessandra Pereira

Eliana Perez

Simone Pettigrew

Fiorella Picchioni

Greg Pierce

Rafael Pinto

Marta Plichta

Ana Poblacion

Ghislain Poda

Maartje Poelman

Lisa Poirier

Michele Polacsek

Jane Polsky

Jennifer Pomeranz

Michael Pomeroy

Francesca Pontin

Elizabeth Pope

Samira Pourmoradian

Behnaz Pourrajab

L. Kirsty Pourshahidi

Shyam Prakash

Sarah Prates

Igor Pravst

Beulah Pretorius

Gabriela Proano

Tyler Prochnow

Jennifer Protudjer

Rachel Prowse

Simone Pröbstl

Claire Pulker

Tirna Purkait

Rafael Pérez-Escamilla

Yanan Qiao

Kathia Quevedo

Matthew Rabbitt

Caroline Racke

Berna Rahi

Mehran Rahimlou

Md. Ashfikur Rahman

Mohammad Mashiur Rahman

Sabuktagin Rahman

Dan Ramdath

Alyssa Ramuscak

Anna Rangan

Madhura Rao

Mylene Ratelle

Neha Rathi

Carola Ray

Jill Reedy

Belinda Reeve

Erica Reeve

David Rehkopf

Clare Relton

Hongqi Ren

Yongcheng Ren

Domenico Rendina

Kerry Renwick

Marcela Reyes

Elise Reynolds

Camila Ricardo

Laurie Ricciuto

Rickelle Richards

Alison Riddle

Chiara Rinaldi

Ana Elisa Rinaldi

Ludivine Ritz

Denise Roberto

Eric Robinson

Brenda Robles

Shannon Robson

Luana Rocha

Michele Rodrigues

Sabine Rohrmann

Jose Rosales Chavez

Anna J. Rose

Emalie Rosewarne

Asher Y. Rosinger

Alexandra Ross

Emily Rousham

Samantha Rowland

Nitai Roy

Cara Ruggiero

Pasquale Rummo

Catherine Russell

Cherie Russell

Joanna Russell

Carrie Ruxton

Samuel Sackar

Shamin Mohd Saffian

Ramiro Salazar Burgos

Amin Salehi-Abargouei

Ana Salinas Martinez

Rosana Salles-Costa

Laura Samuel

Laura J. Samuel

Namrata Sanjeevi

Goncalo Santinha

Francine Santos

Rafaela Santos

Ricardo Santos

Daniel Sanz-Martín

Daniela Sartorelli

Isabela Sattamini

Alice Saul

Tailane Scapin

Peter Scarborough

Ellen Schafer

Rebecca Scharf

Lutz Schomburg

Sally Schultz

Steffen Schulz

Marlene Schwartz

Rebecca Schwei

Carolin Schäfer

Jane Scott

Emmanouela Sdona

Monica Sebastian Kauky

Satheesh Seenivasan

Johannes Seiler

Rini Sekartini

Daniel Sellen

Sakineh Shab-Bidar

Mojtaba Shafiee

Dheeraj Shah

Muhammad Waseem Shah

Muna Shakhshir

Teresa Shamah-Levy

Sarah Shaw

Florence Sheen

Minxue Shen

Farzad Shidfar

Jae Eun Shim

Jee-Seon Shim

Shamarina Shohaimi

Eliza Short

Yurii Shvetsov

Rosely Sichieri

Ewa Sicinska

Alessandro Silva

Andre Silva

Jacqueline Silva

Lara Silva

Scout Silverstein

Cristiane Simoes

Shashi Singh

Susan Sisson

Essi Skaffari

Joyce Slater

Sinne Smed

Falon Smith

Cornelius Smuts

Nadia Sneed

Jakub Sobiecki

Moesijanti Soekatri

Rajan Sonik

Pınar Soylar

Katherine Speirs

Benjamin Spoer

Kelly Squires

Shobhit Srivastava

Muhammad Haroon Stanikzai

Sarah Steele

Simon Steenson

Alexandra Stern

Hayden Stewart

Nelia Steyn

Stefan Storcksdieck genannt Bonsmann

Ron Strochlic

Rocky Strollo

Nanette Ströbele-Benschop

Anna Stubbendorff

Paula Stürmer

Suara Sufyan Bakuri

Minami Sugimoto

Li Sun

Ying Sun

Sana Sungur

Sakari Suominen

Karen Sweazea

Boyd Swinburn

Taren Swindle

Elizabeth Symington

Atakan Tanacan

Hidemi Takimoto

Alison Talbert

Javier Tamargo

Monique Tan

Oana-Adelina Tanasache

Yu-Han Tang

Nattapol Tangsuphoom

Douglas Taren

Md. Tariqujjaman

Ayten A. Tas

Mimi Tatlow-Golden

Pedro Tauler

Francis Tayie

Caroline Taylor

Betzabé Tello

Elisabeth Temme

Fleur Ter Ellen

Ana L. Terry

Alexander Testa

Esther Thatcher

Krisha Thiagarajah

Kittiphong Thiboonboon

Diana Thomas

Dominic Thomas

Preetha Thomas

Tinku Thomas

Claire Thompson

Hannah Thompson

Anne Thorndike

Lukar Thornton

Diane Threapleton

Luu Thi My Thuc

Ornella Tiboni Oschilewski

Anna Timperio

Christiana Rialine Titaley

Patrick Tobi

Manuela Tolmino

Luciana Tomita

Alison Tovar

Dunyaporn Trachootham

Cláudia Tramontt

Lani Trenouth

Thi Huong Trinh

Gina Tripicchio

Helen Truby

Angela C. B. Trude

Emily Truman

Ursula Trübswasser

Marisa Tsai

Zacharie Tsala Dimbuene

Claire N. Tugault-Lafleur

Angelica Tutasi-Lozada

Laurie Twells

Thorkild Tylleskar

Job Ubbink

Ken Uechi

Dr. Kenneth Chinedu Ugoeze

Shigekazu Ukawa

Avita Usfar

Zeynep Uzdil

Chinwe Uzokwe

Rodrigo Valenzuela

Selene Valerino-Perea

Averalda Van Graan

Amber Van den Akker

Duong Van

Jorge Vargas Meza

Nádia Vasconcelos

Juliana Vaz

Gabriela M. Vedovato

Marcela Veiros

Ines Velasco

Alcides Velasquez

Alison Ventura

Henna Vepsäläinen

Pradyuman Verma

Audencio Victor

Helen Vidgen

Bojana Vidovic

Frøydis Nordgård Vik

Lisa Vincze

Marianne Visser

Antonis Vlassopoulos

Rachel Vollmer

Alexander Vonderschmidt

Stephen Vosti

Adam P. Wagner

Simone Wahnschafft

Julia Waity

Claire Wall

Michael Waller

Corinna Walsh

Judd Walson

Karen Walton

Xianlong Wang

Zhenjie Wang

Milkah Wanjohi

Leigh Ward

Lisa Ware

Daniel Warshawsky

Lydiah Waswa

Janet Weber

Jacqueline Webster

M. Margaret Weigel

Hope A. Weiler

Friede Wenhold

André O Werneck

Keith West Jr.

Marianna Wetherill

Shannon Whaley

Lee Wharton

Lauren Whetstone

Susan Whiting

Elizabeth Widen

Michael Widener

Sally Wiggins

Rachel Wilbur

Parke Wilde

Louise Wilson

Megan R. Winkler

Michael Wirth

Julia Wolfson

Julie Wood

Anna Woodhouse

Lauren Woodie

Julie Woods

Charlotte Wright

Summer Wright

Stephanie Wrottesley

Junhui Wu

Qiang Wu

Yangfeng Wu

Sara Wuehler

Lin Xiao

Chao Xuan

Akinori Yaegashi

Siddika Songul Yalcin

Hatice Yalçın

Wai Yew Yang

Wanshui Yang

Yuxiang Yang

Zhenyu Yang

Zahra Yari

Emine Yassıbaş

Pengpeng Ye

Gebretsadik Yehuala

Wan-Ju Yen

Peter Yiga

Xuejun Yin

Yuri Yokoyama

Heng Yaw Yong

Takahiro Yoshizaki

Adrienne Young

Aisha K. Yousafzai

Gamze Yurtdaş Depboylu

Gonca Yıldırım

Gertrude Zeinstra

Lina Zgaga

Junya Zhai

Yiqiang Zhan

Han Zhang

Hongkong Zhang

Ya Zhang

Youjie Zhang

Zhenzhen Zhang

Jian Zhao

Leiyong Zhao

Shuoli Zhao

Zusheng Zheng

Huan Zhou

Qianling Zhou

Hui-lian Zhu

Shankuan Zhu

Ting Zhu

Xu Zhu

Nida Ziauddeen

Meghan Zimmer

Emily Ziraldo

Christina Zorbas

